# Risk Factors of Clonally Related, Multi, and Extensively Drug-Resistant *Acinetobacter baumannii* in Severely Ill COVID-19 Patients

**DOI:** 10.1155/2023/3139270

**Published:** 2023-02-13

**Authors:** Meqdad Saleh Ahmed, Zirak Faqe Ahmed Abdulrahman, Zanan Mohammed Ameen Taha

**Affiliations:** ^1^College of Veterinary Medicine Department of Pathology and Microbiology, University of Duhok, Dahuk, Iraq; ^2^College of Education, Department of Biology, Salahaddin University-Erbil, Erbil, Iraq; ^3^College of Veterinary Medicine Department of Pathology and Microbiology, Duhok Research Center, University of Duhok, Dahuk, Iraq

## Abstract

**Background:**

The secondary infection of multi and extensively drug-resistant “*Acinetobacter baumannii*” in severely ill COVID-19 individuals is usually associated with extended hospitalisation and a high mortality rate. The current study aimed to assess the exact incidence rate of *A. baumannii* coinfection in severely ill COVID-19 patients admitted to intensive care unit (ICUs), to identify the possible mechanism of *A. baumannii* transfer to COVID-19 patients and to find out their resistance rate against different antibiotics.

**Methods:**

Fifty severely ill “COVID-19” individuals on respiratory support were selected with samples being collected from the pharynx. In addition, another 60 samples were collected from the surrounding environment. Bacterial isolates were diagnosed by microbiological cultures and confirmed by “Vitek 2 system” and real-time PCR. The “Vitek 2 Compact system” was used to evaluate these isolates for antimicrobial susceptibility. The recovered isolates' DNA fingerprints and genetic similarities were performed using ERIC-PCR.

**Results:**

Twenty-six samples were tested positive for *A. baumannii* (20 out of 50 samples taken from patients, 40%; 6 out of 60 swabs from a nosocomial setting, 10%). All *A. baumannii* strains isolated from the nosocomial sites were clonally related (have the same genetic lineage) to some strains isolated from patients. However, the majority of the patients' strains were categorised as belonging to the same genetic lineage. Furthermore, “the multi and extensively drug” resistance patterns were seen in all isolates. In addition, total isolates showed resistance to the most commonly tested antibiotics, while none of them was found to be resistant to tigecycline.

**Conclusion:**

Secondary “*A. baumannii*” infection in severely ill “COVID-19” patients is a serious matter, especially when it has one spot of transmission in the ICU as well as when it is extensively drug-resistant, necessitating an immediate and tactical response to secure the issue.

## 1. Introduction

Since the appearance of coronavirus, the world has been in the grasp of coronavirus COVID-19 disease. A large number of patients spend a long time in intensive care units and need invasive mechanical ventilation [1].

Bacterial and fungal superinfections are consequences for patients with viral pneumonia [2]. Nosocomial pneumonia is one of the most common complications for patients' health in the ICU, particularly when ventilated, which might complicate the infection of the lower respiratory tract. Nosocomial infections are generally proven as acquired infections in the patient after 48–72 hours of hospitalisation from admission, and they are mostly spread by devices and instruments and from person to person [3]. Nosocomial infections are mainly caused by many multidrug-resistant (MDR) bacteria; among those, “*A. baumannii*” is one of them [4]. This bacterium can contaminate the environment of the hospital, and it can persist on dry surfaces for a long period of time [5]. As well as, *A. baumannii* isolates are capable of forming biofilms on both “biotic and abiotic” surfaces; hospital facilities and medical equipment are the ideal environments for *A. baumannii* biofilms and hence represent the primary source of infections for patients [6]. Common disinfectants have no effect on it, resulting in outbreaks that are difficult to control and affect the most vulnerable patients in critical condition [7]. During the COVID-19 outbreak, all patients had a chance to acquire a *A. baumannii* infection within at all age groups, even those without underlying diseases [8].

Antibiotic therapy is crucial in the treatment of bacterial “respiratory coinfection,” particularly with multi and extensively drug-resistant *A. baumannii*. This resistance might emerge from extensive misuse of antimicrobial agents in clinical environments [9]. *A. baumannii* is resistant to a large number of antibiotics, including b-lactams, aminoglycosides, fluoroquinolones, and, more recently, carbapenems [10]. When *A. baumannii* becomes resistant to beta-lactams, the last choice will be carbapenems. Though, over the last decades, carbapenem-hydrolyzing-*β*-lactamases of molecular class *B* and *D* have emerged [11]. Class *B* carbapenemases, also termed metallo-beta-lactamases (MBLs), include IMP, VIM, and class *D*, OXA group, which are merged as major carbapenemases in *A. baumannii* [12–14].

For studying *A. baumannii* diversity analysis, molecular typing methods are commonly performed, giving an improvement in molecular biology. Many researchers have applied molecular typing techniques to clinical and environmental *A. baumannii* to study the epidemiological parameters [15]. These include “pulsed-field gel electrophoresis (PFGE) [16], multilocus enzyme electrophoresis (MLEE) [17], and enterobacterial repeated intergenic consensus PCR (ERIC-PCR)” [18]. Furthermore, genome sequencing followed by phylogenetic analysis is also a valuable approach to study antimicrobial resistance and key virulence features of *A. baumannii* isolates [19]. ERIC-PCR is used because it is accurate in making predictions. It is also a quick and easy method that makes epidemiological studies easier for researchers [20].

This is the first study to evaluate “*A. baumannii*” coinfection in “COVID-19 patients” who were admitted to ICUs in a “COVID-19” specialised hospital in Duhok, Iraq. The purpose of this investigation was to determine the exact incidence rate of co-infection by this opportunistic pathogen in severely ill COVID-19 patients, to investigate the suspected mechanism of *A. baumannii* transfer to COVID-19 patients by using the ERIC-PCR assay, and to identify their rate of resistance against different antibiotics that are mainly prescribed for therapeutic purposes in humans.

## 2. Materials and Methods

### 2.1. Study Setting, Ethics, and Participants

Duhok COVID-19 Hospital is a 100-bed hospital in central Duhok city, Iraq. The health ministry of the Kurdistan Region chose this hospital as a specialised “COVID-19” hospital for inpatient support with 2-3 medical emergency chambers, each one containing phasing unit rooms for patients who require respiratory support and ICU nurturing devices. Each room has been updated to seat up to 2-3 “COVID-19” ventilated individuals. In some situations, the number of inpatients may rise by up to 4-5.

This study was cross-sectional and conducted at this specialised “COVID-19” hospital for a period of four months, from August to November 2021 (at the peak of the second wave of the coronavirus outbreak). The general directorate of health (GDH), Duhok, Iraq, approved this study (GDH reference number: 2202021-6-4). All participants in the study or the patient care of the unconscious ones gave their permission (an informed consent form was signed by each patient or their relative). The form included the criteria about the importance of secondary bacterial infection after COVID-19 infection as the main cause of death, as well as the fact about which effective antibiotics can be prescribed in COVID-19 cases.

The participants in this study were 50 severely ill patients (gender patients were 28 male and 22 female, while the age groups were ≤40 years, 41–60 years, and >60 years) hospitalised in ICU wards in specialised COVID-19 hospitals. Each patient included in this investigation was admitted to the hospital, infected with COVID-19 (confirmed cases by qPCR from the hospital laboratory), mechanically ventilated, and intubated for more than 48 hours in ICUs. Furthermore, a temperature >38°C or hypothermia of 36°C, “purulent tracheal secretions, a reduction in PaO_2_/FiO_2_,” and increasing infiltration on the chest radiograph were additionally noted. Furthermore, all participants were “neutropenic,” with a high “erythrocyte sedimentation rate and C-reactive protein,” as well as symptoms of throat infection, coughing, and breathing difficulty. Corticosteroids were administered to all patients with severe decreases in the PaO_2_/FiO_2_ value, and also, they were commonly given to patients with high serum levels of interleukins. In addition, all patients have been prescribed an intravenous infusion or intramuscular injection of carbapenems or other broad-spectrum antibiotics, such as azithromycin, clarithromycin, ceftriaxone, erythromycin, amoxicillin, ciprofloxacin, and levofloxacin, at admission as prophylactic use. All of the above criteria were obtained from the hospital records.

### 2.2. Sample Collection and *A. baumannii* Detection

A total of 110 swabs were taken from “COVID-19” individuals who were severely ill. Pharyngeal swabs from the COVID-19 hospital, as well as the surrounding nosocomial ICU equipment and environments (50/60, respectively). A pharyngeal swab was obtained under complete aseptic conditions with the correct use of personal protective equipment (PPE). Swabs were moistened in sterile normal saline and wrapped around the ICU equipment and surrounding patient environments for nosocomial sampling (external and internal surfaces of the noninvasive ventilation mask, entire surfaces of the mechanical ventilator screen, and surfaces of the ICU bed railing). For safety precautions and preventing the “COVID-19” virus from spreading far outside the hospital, the collected swabs were directly cultured in the hospital microbiology laboratory on CHROMagar™ *Acinetobacter* (Chromagar, France) under strict aseptic conditions. After that, all plates were transported to the microbiology laboratory in the College of Veterinary Medicine and incubated at 37°C for 18–24 h. The presumed red colonies were cultivated on MacConkey agar and incubated as previously described. Colonies that do not ferment the lactose (pale yellow) were confirmed as *A. baumannii* using the VITEK®2 compact system (Bio-Mérieux, France) using the “Vitek 2 GN ID Card (Gram-Negative Identity Card). The identification protocol by the VITEK®2 system was made according to the manufacturer's recommendations. The final confirmation of isolates was performed using a real-time PCR assay to amplify 16S–23S ribosomal DNA. The stock preparations were done in brain heart infusion broth (Lab M, UK) with 25% (v/v) glycerol added. The broth was kept at −20°C [21].

### 2.3. DNA Extraction and Molecular Detection of *A. baumannii* Isolates

According to Garcia and his colleagues [22], DNA specimens were separated using the thermal separation technique. Subsequently, 150 *μ*l of the supernatant was conserved to be utilised as a “PCR DNA” template. A “nanodrop (Thermo Scientific, USA)” was used to measure the concentration and quality of isolated DNA.

For the detection of *A. baumannii*, real-time PCR was performed based on the identification of 16S–23S ribosomal DNA using primers; forward 5′ CATTATCACGGTAATTAGTG and reverse 5′ AGAGCACTGTGCACTTAAG. The PCR cycler was set up in a qPCR Bio-Rad CFX96 (BIO-RAD/Germany) as follows: 95 C for 3 min as initial denaturation, followed by 30 cycles of 95°C for 45 min, 62°C for 45 min, 72°C for 45 min [23]. *Acinetobacter baumannii* and *Klebsiella pneumonia* (obtained from the Duhok Research Centre at the College of Veterinary Medicine, University of Duhok) were used as a positive and negative control, respectively, with each PCR reaction. The PCR mixtures were carried out in a 20 *μ*l volume comprising of 10 *μ*l Sybr green master mix (Add SYBR Master from Addbio/Korea), 1 *μ*l of each set of primers (10 pmol/*μ*l for each primer), 1 *μ*l of sample DNA (50–100 ng/*μ*l), and 7 *μ*l DNase/RNase-free water.

### 2.4. Clonal Relatedness and Diversity Analysis

“ERIC-PCR” was utilised to detect related strains of *A. baumannii* and identify variants, utilising sequences of primers “(ERIC1: 5′-ATGTAAGCTCCTGGGGATTCAC-3′ and ERIC2: 5′-AAGTAAGTGACTGGGGTGAGCG-3′)” previously designated by Saleem and his colleagues [24]. The ERIC-PCR method was performed in a total volume of 25 *μ*l, containing 2 *μ*l of each primer (10 pmol/*μ*l), 12.5 *μ*l of “hot start master mix” (AddBio, Korea), 2 *µ*l of DNA with a concentration of 30–100 ng/*µ*l, and 6.5 *µ*l of “nuclease-free water (Qiagen, Germany)” [25]. The PCR was performed using the “PCR system 9700 GeneAmp (Applied Biosystem, USA),” and according to Bakhshi et al. [26], the “PCR setting” was utilised. The initial denaturation was 94°C for 5 minutes, followed by 35 cycles of 94°C for 1 minute, 1 minute at 54°C, then 8 minutes at 72°C, with a final extension of 10 minutes at 72°C [25]. Finally, the PCR products were put into a 2% agarose with “1X Tris-acetate-EDTA (TAE) buffer” and labelled with a “red safe DNA staining solution (GeNetBio, Korea).” A DNA ladder “(100-bp DNA ladder/Genedirex, Taiwan)” was used. Finally, for ERIC-PCR data analysis, a photograph was captured of the gel.

### 2.5. Data Processing for ERIC-PCR

An electrophoresis picture containing 26 isolates of *A. baumannii* was first observed for the DNA band's existence or absence in the ERIC-PCR gel, and then, by “GelJ software version 2.0 (available at https://sourceforge.net/projects/gelj/),” the dendrogram was generated. The genotyping of the strains was created using the “Unweighted Pair Group Method with Arithmetic Mean (UPGMA)” methodology based on the Dice similarity coefficient with a tolerance of 2% [27]. Electrophoresis patterns with a similarity coefficient of at least 80% (similarity limits of 80% or above) were grouped with the same genotypes or the same group in ERIC-PCR [28]. Strains were clustered together based on their sample source (either COVID-19 patients or the surrounding nosocomial ICU equipment and environments).

### 2.6. Antimicrobial Susceptibility Testing (AST)

The confirmed isolates by conventional culture, VITEK 2 system, and real-time PCR were subjected to antimicrobial susceptibility testing against 33 antibiotics (Table 1) by the automated Vitek 2 Compact system (VITEK® 2 system, BioMariex, France) [41] and by the standard disc diffusion method on “Mueller-Hinton agar (Lab M, UK).” Antibiotics were chosen to cover as much as possible to achieve the best therapeutic options. The methodology for the determination of inhibition zone breakpoints was followed by the authors of [42, 43]. Isolates were marked as either susceptible or resistant. The intermediate susceptible isolates to the specific antibiotic were classified as resistant using guidelines from the clinical and laboratory standards institute (CLSI, 2012). Samples that were initially susceptible or intermediately resistant to one antibiotic may become resistant after therapy. Then, any isolate in this study was classified as a resistant isolate to antibiotics when it was intermediately resistant to specific antibiotics [44]. Any isolate that was resistant to three or more antibiotics was known as multiple antibiotic resistance (MAR). In contrast, isolate was resistant to at least one agent in all antibiotic groups; however, two or fewer antimicrobial susceptible were categorised as extensively drug-resistant (XDR) [45].

### 2.7. Statistical Analysis

All datasets were analysed by one-way analysis of variance (ANOVA). Specific differences between the groups were determined using the Duncan multiple range test using SPSS version 20. The accepted level of significance was *P* ≤ 0.05.

## 3. Results

### 3.1. The Incidence of *Acinetobacter baumannii*

**Figure 1:**
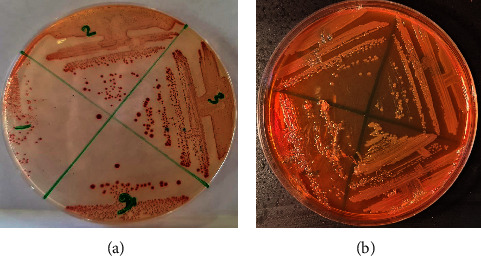
(a) Red colonies of *A. baumannii* on CHROMagar™ Acinetobacter agar. (b) Nonlactose fermenter colonies of *A. baumannii* on MacConkey agar.

**Figure 2:**
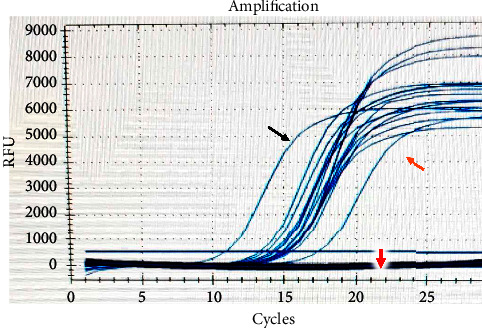
Real-time PCR detection of *A. baumannii*. The black arrow represents the positive control, the orange arrow represents the samples, and the red arrow represents the negative control.

One hundred ten samples, collected from the ICU of the COVID-19 hospital, were analysed by conventional microbiological and molecular assays for the detection of *A. baumannii*. Conventional methods of isolation for *Acinetobacter* species, including red colonies on chromogenic agar (CHROMagar™ Acinetobacter) (Figure 1(a)) and nonlactose fermenter colonies on MacConkey agar (Figure 1(b)), revealed that 20 isolates (40%) were positive out of 50 samples taken from patients. In comparison, 6 positive isolates were collected from the other 60 nosocomial samples (10%). All 26 positive samples were tested by the VITEK®2 compact system (Bio-Mérieux, France) using the Vitek 2 GN ID Card (Gram-Negative Identity Card), then confirmed by a “real-time PCR” assay (Figure 2), and all isolates were found to be *A. baumannii*. In this study, all samples were positive for both methods, and there were no differences between them.

Out of 20 *A. baumannii* isolates from patients, 11 (55%) were from males, and 9 (45%) were from females. The median age of patients with *A. baumannii* infection was 52.5 years (range: 21–84 years), with 13 (65%) of the isolates coming from patients aged 41–60 years (8; 61.5% male and 5; 38.5% female); 5 patients (25%) aged over 60 years (2; 40% male and 3; 60% female); and 2 (10%) from patients 40 years (1; 50% male and 1; 50% female). *Acinetobacter baumannii* was significantly observed in male patients in the age group of 41–60 years (*P* ≤ 0.005). At the same time, there was no statistical correlation in the total incidence rate between genders (Table 2).

### 3.2. Molecular Typing of *Acinetobacter baumannii* by ERIC-PCR

**Figure 3:**
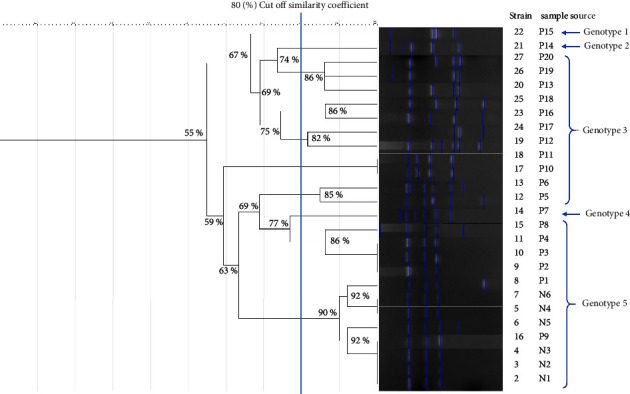
The banding pattern of 26 *A. baumannii* strains isolated from the ICU environment and COVID-19 individuals is shown in a dendrogram obtained from ERIC-PCR. *P*: patient, *N*: nosocomial (ICU environment).

The outcome showed that based on the number and size of ERIC sequence variances and depending on the ERIC-PCR fingerprinting analysis that was seen in each isolate, the *A. baumannii* isolate similarity was between 55–100%. The strains were divided into five genotypes based on a similarity limit of 80% (1–5), in which the most prevalent clones were genotypes 3 and 5 and their variants among the isolates, including 23/26; 88.4% of total isolates (Figure 3, Table 3). Genotype 5 was the largest group, containing 12 strains, including 6 isolates from the ICU environment, 2 from the patients ≤40 years of age group (1 male and 1 female), and 4 from the patients 41–60 years of age group (3 males and 1 female) while 11 strains clustered in genotype 3, comprised of 7 patients from the 41–60 age group (3 males and 4 females) and 4 patients from the age group >60 years (2 males and 2 females). In contrast, genotypes 1, 2, and 4 were comprised of only one strain. Interestingly, most of the diversity of strains were seen in the male 41–60 age group (Table 4). Our results showed that all strains recovered from nosocomial sites had the same genetic similarity to some strains obtained from patients (the band profile of isolates within the nosocomial setting showed a cluster similarity to 6 representatives of the patient's *A. baumannii* isolates). With regards to the patient's strains, most were clustered with the same genotype (same genetic lineage), including strain numbers 27, 28, 20, 25, 23, 24, 19, 18, 1,7, 13, and 12 (Figure 3, Table 3).

### 3.3. Susceptibility Testing for Antibiotics

**Figure 4:**
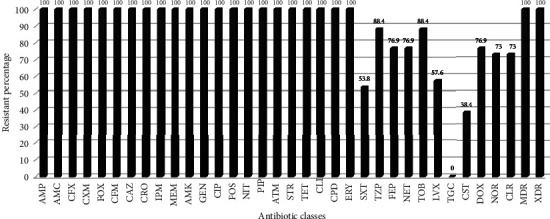
Total antibiotic resistance percentage to *Acinetobacter baumannii* isolates from both patients and nosocomial.

All strains were found to be 100% resistant to at least three or more antibiotics (100% were MAR), and all of them showed nonsusceptibility to at least one agent in all antibiotic groups but two or fewer antimicrobials (100% were XDR). The resistance patterns to the tested antibiotics were as follows: all of them showed a total resistance (100%) to each of ampicillin, amoxicillin/clavulanate, cefuroxime, cefuroxime axetil, cefoxitin, cefixime, ceftazidime, ceftriaxone, imipenem, meropenem, amikacin, gentamicin, ciprofloxacin, fosfomycin, nitrofurantoin, piperacillin, aztreonam, streptomycin, tetracycline, clindamycin, cefpodoxime, and erythromycin, followed by piperacillin-tazobactam and tobramycin each of about (88.4%), (76.9%) of strains exhibited resistant for doxycycline, cefepime, and netilmicin, (73%) of isolates were resistant to norfloxacin and clarithromycin, (57.6%) by levofloxacin, (53.8%) by trimethoprim-sulfamethoxazole. This was followed by (38.4%) resistance to colistin; the mean minimum inhibitory concentration (MIC) value for colistin-resistant was ≥16 *μ*g/mL. All strains displayed a total susceptibility to tigecycline (Table 5, Figure 4). With regard to the age groups, the resistance rate of *A. baumannii* in patients 41–60 years old showed a higher resistance rate (total resistant pattern) than the others (*P* ≤ 0.05). While there were no significant differences were found between genders, except TOB and NET in isolates from females were found to be significantly resistant (*P* ≤ 0.05) (Table 6).

## 4. Discussion

Secondary bacterial infection is one of the neglectable issues in severe and critical COVID-19 patients. In this study, risk factors for *A. baumannii* coinfection in COVID-19 patients were looked at by gender, age, and how the infection was spread and how resistant it was to antibiotics.

Our finding shows a high incidence rate of *A. baumannii* coinfection. This could be due to the persistent environmental contamination in the ICU setting, which in turn can be attributed to factors prompting the quality of care provided. Furthermore, the incidence of ICU-acquired infections, such as ICU type, used equipment rate, and admission/discharge criteria, at the peak of coronavirus infection, could be another factor attributed to this situation [42, 46]. On the other hand, any patient with a viral infection may have increased susceptibility to bacterial coinfection [46]. This is typically accomplished through detrimental epithelial ciliary clearance and immunological dysfunction [47]. Furthermore, corticosteroids that inhibit IL-6 may have opposing negative effects on “innate immune responses” and microbial clearance [48]. Moreover, it has recently been shown that *A. baumannii* invade pneumocytes by targeting “human carcinoembryonic antigen-related cell adhesion molecules (CEACAMs).” This indicates that “CEACAM” overexpression may increase the risk of infection of the lower respiratory tract, specifically in “severely ill COVID-19 patients” [49].

Concerning the incidence rate of *A. baumannii* coinfection in relation to age and gender, a high incidence was significantly seen in male patients in the age group of 41–60 years. As a result, men seem to be more susceptible to COVID-19 [50], especially those in the active working age group, being outside more frequently due to working conditions, leading to a high rate of COVID-19 infection that increases the incidence of coinfection with *A. baumannii* [51]. It could also be due to the difference in hormonal status between men and women, which causes females to have a stronger immune response to microbes than males, resulting from the variation in hormonal status among genders (testosterone has anti-inflammatory effects that reduce the immune response to infection) [52].

Regarding the ERIC-PCR results, a clonal spread of *A. baumannii* strains between the ICU setting and the COVID-19 patients was found. These data suggest that the same clones were circulating within the hospital, which means that the source of *A. baumannii* infection is the hospital ICU's environment itself. *Acinetobacter baumannii* can withstand for long periods on various surfaces against hard environmental conditions supported by the biofilm formation potential [53]. This may facilitate the cross-transmission between ICU equipment and COVID-19 patients. To support this hypothesis, Shinohara and his colleagues found a 100% genetic similarity between all *A. baumannii* strains collected from COVID-19 patients and the ICU equipment and the surrounding environment [54]. In contrast, some patients' strains were clustered with the same genotype, indicating that there was a cross-transmission of *A. baumannii* between these patients.

We did not find any specific reason behind the diverse appearance of strains isolated from patients in the age group of 41–60 years; however, it may be due to the large number of *A. baumannii*-infected patients who belong to this age group.

Pathogen identification and antimicrobial susceptibility test methods are critical for commencing effective medication and preventing further complications [55]. The high rate of resistance to antibiotics in this investigation may be linked to prolonged exposure to antibiotics, and this may be due to the long duration of hospitalisation in the ICU [56]. In addition, with COVID-19 patients, most antibiotics were empirical [57], and there was extensive improper use of antibiotics [28].

With regarding the multi and extensively drug-resistant “*Acinetobacter baumannii*,” this research demonstrated the clustering of distinct-resistant genes within the same genetic component and the coselection pathway of the resistant or by mutation of specific genes that were usually extruding a wide variety of drugs, mostly due to the expression of a gene which codes for multiple drug efflux pumps [58]. Efflux pump inhibitor drugs have shown promising results in a number of studies, which gives hope that MDR *A. baumannii* resistance can be overcome [59, 60].

Carbapenem resistance in *A. baumannii* (CRAb) is a major issue since this category of antimicrobial is used to treat infections caused by multidrug-resistant Gram-negative bacteria as the last line of defense [61]. The study's outcome showed that all isolates were carbapenem-resistant. Reflecting the higher patient exposure to these drugs, as in most parts of the world, the drug of choice in severely ill COVID-19 patients to prevent secondary bacterial infection is imipenem or meropenem [62].

There is a second-line treatment strategy to compensate for CRAb infections, including tigecycline and polymyxins [63]. Even though tigecycline has never been used as a therapy in our area, our isolates showed that they were completely susceptible to it, and this will create hope of treating coinfection with CRAb in COVID-19 patients.

On the other hand, some isolates were resistant to colistin, making the selection of a rational antimicrobial regimen extremely difficult. Colistin resistance is either caused by the horizontally transferable colistin-resistant genes or through mutations of genes in clusters that respond to colistin stress and encode proteins that are involved in lipopolysaccharide biosynthesis pathways [64] with high MIC levels [65]. Some of the *A. baumannii* isolates were colistin-resistant (there were no outbreaks of colistin resistance), suggesting the mechanism of colistin resistance may have arisen in these isolates by mutational changes.

In this study, there was no gender-based difference in the frequency of antibiotic resistance, except for TOB and NET in females, which were found to be significantly resistant (*P* < 0.05). This could be due to antibiotic-resistant pressure resulting from increased previous exposure to these drugs against the most frequent bacterial infections in females [66], such as urinary tract infections [67], while, for age groups, the resistance rate of *A. baumannii* from the patients 41–60 years old showed a higher resistance rate than the others. This might be attributed to the high incidence rate of infection among this age group due to the large number of COVID-19 patients in hospitals who belong to this age group.

These facts might not apply to all regions because the prevalence and susceptibilities of bacterial resistance differ widely among COVID-19 pandemics in different geographical regions. Therefore, a local survey is required on antibiotic susceptibilities to estimate these results.

Finally, the present work has strongly recommended a complete hygienic condition to be implemented in the ICUs, with the effective application of COVID-19 aseptic techniques, including protective clothing and equipment, and environmental disinfection measures, to restrict the transmission of bacterial infection. In addition, it is preferable to use the most effective or new antibiotics with different modes of action to defeat the growth of MDR *A. baumannii* in COVID-19 bacterial coinfection.

## 5. Conclusion

The appearance of COVID-19 disease caused by the coronavirus increases the risk of coinfection by multidrug-resistant bacteria. *Acinetobacter baumannii* is one of the life-threatening MDR bacteria that causes superinfection in severely ill COVID-19 patients. This study highlights a high incidence rate of genetically related *A. baumannii,* especially in active age group male patients. Hence, it emphasises standard preventive strategies to minimise further bacterial spread among COVID-19 patients. As a consequence of XDR *A. baumannii*, the therapeutic option for this bacterium becomes challenging. However, there is some hope to treat this extensively drug-resistant *A. baumannii* with tigecycline. Therefore, it is recommended to perform antibiotic susceptibility testing in COVID-19 patients suffering from secondary bacterial infections to prevent the empirical prescription of antibiotics, with a subsequent decrease in resistance development.

## Figures and Tables

**Table 1:** The classes of antibiotics used in this study and their mechanisms of action were obtained from previous studies.

Class of antibiotics	Antibiotics	Mechanism of action	References
Penicillin	Ampicillin, piperacillin, piperacillin-tazobactam, and cefepime	Disrupt the synthesis of the peptidoglycan layer of bacterial cell walls	[34]
Penicillin combinations	Amoxicillin/clavulanate	The second component reduces the effectiveness of some forms of bacterial resistance to the first component	[35]
Cephalosporins	Cefuroxime 2^nd^ generation, cefoxitin 2^nd^ generation, cefixime 3^rd^ generation, ceftazidime 3^rd^ generation, ceftriaxone 3^rd^ generation, cefpodoxime 2^nd^ generation	Disrupt the synthesis of the peptidoglycan layer of bacterial cell walls	[36]
Carbapenems	Imipenem, meropenem	Inhibition of cell wall synthesis	[34]
Aminoglycosides	Amikacin, gentamicin, netilmicin, tobramycin, streptomycin	It inhibits the translocation of the peptidyl-tRNA from the A-site to the P-site, leaving the bacterium unable to synthesize proteins vital to its growth	[37]
Quinolones/fluoroquinolones	Ciprofloxacin, levofloxacin, norfloxacin	Inhibits the bacterial DNA gyrase or the topoisomerase IV enzyme, thereby inhibiting DNA replication and transcription	[34]
Fosfomycin	Fosfomycin	Inactivates enol pyruvyl transferase, thereby blocking cell wall synthesis	[38]
Nitrofurans	Nitrofurantoin	Reduced by bacterial flavoproteins to reactive intermediates that inhibit bacterial ribosomes and other macromolecules	[39]
Sulfonamides	Trimethoprim-sulfamethoxazole	Folate synthesis inhibition	[40]
Monobactams	Aztreonam	Disrupt the synthesis of the peptidoglycan layer of bacterial cell walls	[41]
Tetracyclines	Tetracycline, doxycycline	Inhibits the binding of aminoacyl-tRNA to the mRNA-ribosome complex. They do so mainly by binding to the 30S ribosomal subunit in the mRNA translation complex	[34]
Glycylcycline	Tigecycline	It has a similar structure and action to tetracycline, but it is stronger	[42]
Polymyxin	Colistin	Blocks the peptidoglycan bacterial cell wall outside of the inner membrane	[43]
Lincosamides	Clindamycin	Binds to the 50S subunit of bacterial ribosomal RNA thereby inhibiting protein synthesis	[44]
Macrolides	Clarithromycin, erythromycin	Inhibition of bacterial protein biosynthesis by binding reversibly to the subunit 50S S of the bacterial ribosome, thereby inhibiting translocation of peptidyl-tRNA	[45]

**Table 2:** Distribution of *A. baumannii* between age groups and gender.

Age group	*n* (%) isolates	Isolates by gender (%)	
		Male	Female
≤40 years, *n* = 9	2 (10)	1 (50)	1 (50)
41–60 years, *n* = 24	13 (65)	8 (61.5)^*∗*^	5 (38.5)
>60 years old, *n* = 17	5 (25)	2 (40)	3 (60)
Total 50	20 (40)	11 (55)	9 (45)

^*∗*^indicates that *A. baumannii* was significantly seen in male patients (41–60 years age group) at *P* ≤ 0.005.

**Table 3:** The genotypic pattern of 26 *A. baumannii* strains isolated from the ICU environment and severely ill COVID-19 patients.

Strain number	Sample source	Gender	Age range (years)	Genotypic pattern
22	Patient 15	*M*	41–60	Genotype 1
21	Patient 14	*F*	>60	Genotype 2
27	Patient 20	*F*	41–60	Genotype 3
28	Patient 19	*M*	>60	
20	Patient 13	*M*	41–60	
25	Patient 18	*F*	>60	
23	Patient 16	*F*	41–60	
24	Patient 17	*M*	>60	
19	Patient 12	*F*	>60	
18	Patient 11	*M*	41–60	
17	Patient 10	*F*	41–60	
13	Patient 6	*F*	41–60	
12	Patient 5	*M*	41–60	
14	Patient 7	*M*	41–60	Genotype 4
15	Patient 8	*F*	41–60	Genotype 5
11	Patient 4	*M*	41–60	
10	Patient 3	*F*	≤40	
9	Patient 2	*M*	≤40	
8	Patient 1	*M*	41–60	
7	Nosocomial 6			
5	Nosocomial 4			
6	Nosocomial 5			
16	Patient 9	*M*	41–60	
4	Nosocomial 3			
3	Nosocomial 2			
2	Nosocomial 1			

**Table 4:** Genotypic frequency of *A. baumannii* according to age groups and gender.

Genotypes	Age/gender					
	≤40 years		41–60 years		>60 years	
	Male	Female	Male	Female	Male	Female
Genotypes 1	0	0	1	0	0	0
Genotypes 2	0	0	0	0	0	1
Genotypes 3	0	0	3	4	2	2
Genotypes 4	0	0	1	0	0	0
Genotypes 5^*∗*^	1	1	3	1	0	0
Total	1	1	8	5	2	3

^*∗*^All 6 nosocomial isolates were excluded.

**Table 5:** Antibiotic resistance pattern to *Acinetobacter baumannii* isolates.

Antibiotics	Patients (#20) no (%)		Nosocomial (#6) no. (%)		Total resistant (#26) no. (%)
	*R*	*S*	*R*	*S*	
AMP	20 (100)	0 (0)	6 (100)	0 (0)	26 (100)
AMC	20 (100)	0 (0)	6 (100)	0 (0)	26 (100)
CFX	20 (100)	0 (0)	6 (100)	0 (0)	26 (100)
CXM	20 (100)	0 (0)	6 (100)	0 (0)	26 (100)
FOX	20 (100)	0 (0)	6 (100)	0 (0)	26 (100)
CFM	20 (100)	0 (0)	6 (100)	0 (0)	26 (100)
CAZ	20 (100)	0 (0)	6 (100)	0 (0)	26 (100)
CRO	20 (100)	0 (0)	6 (100)	0 (0)	26 (100)
IPM	20 (100)	0 (0)	6 (100)	0 (0)	26 (100)
MEM	20 (100)	0 (0)	6 (100)	0 (0)	26 (100)
AMK	20 (100)	0 (0)	6 (100)	0 (0)	26 (100)
GEN	20 (100)	0 (0)	6 (100)	0 (0)	26 (100)
CIP	20 (100)	0 (0)	6 (100)	0 (0)	26 (100)
FOS	20 (100)	0 (0)	6 (100)	0 (0)	26 (100)
NIT	20 (100)	0 (0)	6 (100)	0 (0)	26 (100)
PIP	20 (100)	0 (0)	6 (100)	0 (0)	26 (100)
ATM	20 (100)	0 (0)	6 (100)	0 (0)	26 (100)
STR	20 (100)	0 (0)	6 (100)	0 (0)	26 (100)
TET	20 (100)	0 (0)	6 (100)	0 (0)	26 (100)
CLI	20 (100)	0 (0)	6 (100)	0 (0)	26 (100)
CPD	20 (100)	0 (0)	6 (100)	0 (0)	26 (100)
ERY	20 (100)	0 (0)	6 (100)	0 (0)	26 (100)
SXT	10 (50)	10 (50)	4 (66.6)	2 (33.3)	14 (53.8)
TZP	18 (90)	2 (10)	5 (83.3)	1 (16.6)	23 (88.4)
FEP	16 (80)	4 (20)	4 (66.6)	2 (33.3)	20 (76.9)
NET	16 (80)	4 (20)	4 (66.6)	2 (33.3)	20 (76.9)
TOB	18 (90)	2 (10)	5 (83.3)	1 (16.6)	23 (88.4)
LVX	12 (60)	8 (40)	3 (50)	3 (50)	15 (57.6)
TGC	0 (0)	20 (100)	0 (0)	6 (100)	(0)
CST	8 (40)	12 (60)	2 (33.3)	4 (66.6)	10 (38.4)
DOX	15 (75)	5 (25)	5 (83.3)	1 (16.6)	20 (76.9)
NOR	15 (75)	5 (25)	4 (66.6)	2 (33.3)	19 (73)
CLR	14 (70)	6 (30)	5 (83.3)	1 (16.6)	19 (73)
MDR	20 (100)	0 (0)	6 (100)	0 (0)	26 (100)
XDR	20 (100)	0 (0)	6 (100)	0 (0)	26 (100)

AMP: ampicillin, AMC: amoxicillin/clavulanate, CFX: cefuroxime, CXM: cefuroxime axetil, FOX: cefoxitin, CFM: cefixime, CAZ: ceftazidime, CRO: ceftriaxone, IPM: imipenem, MEM: meropenem, AMK: amikacin, GEN: gentamicin, CIP: ciprofloxacin, FOS: fosfomycin, NIT: nitrofurantoin, SXT: trimethoprim-sulfamethoxazole, PIP: piperacillin, TZP: piperacillin-tazobactam, FEP: cefepime, ATM: aztreonam, NET: netilmicin, TOB: tobramycin, LVX: levofloxacin, STR: streptomycin, TET: tetracycline, TGC: tigecycline, CST: colistin, CLI: clindamycin, CPD: cefpod-oxime, DOX: doxycycline, NOR: norfloxacin, CLR: clarithromycin, ERY: erythromycin. MDR: multidrug-resistant, XDR: extensively drug resistance, *S*: suspectable, *R*: resistant.

**Table 6:** Antibiotic resistance pattern of *A. baumannii* in relation to gender and age groups.

Antibiotics	Gender			Age group		
	Total isolates *n* = 20 (%)	Male isolates no = 11 (%)	Female isolates no = 9 (%)	≤40 no = 2 (%)	41–60 no = 13 (%)	>60 no = 5 (%)
AMP, AMC, CFX, CXM, FOX, CFM, CAZ, CRO, IPM, MEM, AMK, GEN, CIP, FOS, NIT, PIP, ATM, STR, TET, CLI, CPD, ERY	20 (100)	11 (100)	9 (100)	2 (100)	13 (100)	5 (100)
TZP	18 (90)	10 (91)	8 (88.9)	2 (100)	11 (84.6)	5 (100)
TOB	18 (90)	9 (81.8)	9 (100)^*∗∗*^	2 (100)	11 (84.6)	5 (100)
FEP	16 (80)	9 (81.8)	7 (77.7)	1 (50)	10 (77)	5 (100)
NET	16 (80)	7 (54.5)	9 (100)^*∗∗*^	1 (50)	10 (77)	5 (100)
DOX	15 (75)	8 (72.7)	7 (77.7)	2 (100)	9 (69.2)	4 (80)
NOR	15 (75)	9 (81.8)	6 (66.6)	1 (50)	9 (69.2)	5 (100)
CLR	14 (70)	10 (91)	4 (44.4)	1 (50)	8 (61.5)	5 (100)
SXT	10 (50)	4 (36.4)	6 (66.6)	0 (0)	6 (46.1)	4 (80)
CST	8 (40)	5 (45.4)	3 (33.3)	0 (0)	5 (38.4)	3 (60)
Total resistant pattern	150 (100)	82 (54.6)	68 (45.4)	12 (8)	92 (61.4)^*∗*^	46 (30.6)

^*∗*^indicates that the total resistant pattern of *A. baumannii* from the age group of 41–60 years was significantly higher than the other age groups (*P* ≤ 0.05). ^*∗∗*^TOB and NET in females were found to be significantly resistant (*P* ≤ 0.05).

## Data Availability

No data were used to support this study.

## References

[1] Goyal P., Choi J. J., Pinheiro L. C. (2020). Clinical characteristics of Covid-19 in New York city. *New England Journal of Medicine*.

[2] Paparoupa M., Aldemyati R., Roggenkamp H. (2022). The prevalence of early-andlate-onset bacterial, viral, and fungal respiratory superinfections in invasively ventilated COVID-19 patients. *Journal of Medical Virology*.

[3] Agaba P., Tumukunde J., Tindimwebwa J. V. B., Kwizera A. (2017). Nosocomial bacterial infections and their antimicrobial susceptibility patterns among patients in Ugandan intensive care units: a cross sectional study. *BMC Research Notes*.

[4] Dandagi G. L. (2010). Nosocomial pneumonia in critically ill patients. *Lung India: Official Organ of Indian Chest Society*.

[5] Nutman A., Lerner A., Schwartz D., Carmeli Y. (2016). Evaluation of carriage and environmental contamination by carbapenem-resistant Acinetobacter baumannii. *Clinical microbiology and infection*.

[6] Pompilio A., Scribano D., Sarshar M., Di Bonaventura G., Palamara A. T., Ambrosi C. (2021). Gram-negative bacteria holding together in a biofilm: the Acinetobacter baumannii way. *Microorganisms*.

[7] Peleg A. Y., Seifert H., Paterson D. L. (2008). Acinetobacter baumannii: emergence of a successful pathogen. *Clinical Microbiology Reviews*.

[8] Sharifipour E., Shams S., Esmkhani M. (2020). Evaluation of bacterial co-infections of the respiratory tract in COVID-19 patients admitted to ICU. *BMC Infectious Diseases*.

[9] Rangel K., Chagas T. P. G., De-Simone S. G. (2021). Acinetobacter baumannii infections in times of COVID-19 pandemic. *Pathogens*.

[10] Sarshar M., Behzadi P., Scribano D., Palamara A. T., Ambrosi C. (2021). Acinetobacter baumannii: an ancient commensal with weapons of a pathogen. *Pathogens*.

[11] Shahcheraghi F., Abbasalipour M., Feizabadi M., Ebrahimipour G., Akbari N. (2011). Isolation and genetic characterization of metallo-*β*-lactamase and carbapenamase producing strains of Acinetobacter baumannii from patients at Tehran hospitals. *Iranian Journal of Microbiology*.

[12] Behzadi P., Garcia-Perdomo H. A., Karpinski T. M., Issakhanian L. (2020). Metallo-ß-lactamases: a review. *Molecular Biology Reports*.

[13] Walsh T. R., Toleman M. A., Poirel L., Nordmann P. (2005). Metallo-*β*-lactamases: the quiet before the storm?. *Clinical Microbiology Reviews*.

[14] Feizabadi M. M., Fathollahzadeh B., Taherikalani M. (2008). Antimicrobial susceptibility patterns and distribution of blaOXA genes among Acinetobacter spp. Isolated from patients at Tehran hospitals. *Japanese Journal of Infectious Diseases*.

[15] Ceparano M., Baccolini V., Migliara G. (2022). Acinetobacter baumannii isolates from COVID-19 patients in a hospital intensive care unit: molecular typing and risk factors. *Microorganisms*.

[16] Mohammadi Bardbari A., Mohajeri P., Arabestani M. R. (2020). Molecular typing of multi-drug resistant Acinetobacter baumannii isolates from clinical and environmental specimens in three Iranian hospitals by pulsed field gel electrophoresis. *BMC Microbiology*.

[17] Gaiarsa S., Batisti Biffignandi G., Esposito E. P. (2019). Comparative analysis of the two Acinetobacter baumannii multilocus sequence typing (MLST) schemes. *Frontiers in Microbiology*.

[18] Sepahvand S., Darvishi M., Mokhtari M., Ali Davarpanah M. (2022). Evaluation of genetic diversity of colistin-resistant Acinetobacter baumannii by BOX-PCR and ERIC-PCR: the first report. *Future Microbiology*.

[19] Marazzato M., Scribano D., Sarshar M. (2022). Genetic diversity of antimicrobial resistance and key virulence features in two extensively drug-resistant acinetobacter baumannii isolates. *International Journal of Environmental Research and Public Health*.

[20] Karimi Z., Ahmadi A., Najafi A., Ranjbar R. (2018). Bacterial CRISPR regions: general features and their potential for epidemiological molecular typing studies. *The Open Microbiology Journal*.

[21] Taha Z. M. (2021). Genetic diversity and clonal relatedness of Aeromonas hydrophila strains isolated from hemorrhagic septicemia’s cases in common Carp (Cyprinus carpio) farms. *Iraqi Journal of Veterinary Sciences*.

[22] García-Meniño I., Forcelledo L., Rosete Y., Garcia-Prieto E., Escudero D., Fernandez J. (2021). Spread of OXA-48-producing Klebsiella pneumoniae among COVID-19-infected patients: the storm after the storm. *Journal of infection and public health*.

[23] Ghaffoori Kanaan M. H., Al-Shadeedi S. M., Al-Massody A. J., Ghasemian A. (2020). Drug resistance and virulence traits of Acinetobacter baumannii from Turkey and chicken raw meat. *Comparative Immunology, Microbiology and Infectious Diseases*.

[24] Saleem S., Bokhari H. (2020). Resistance profile of genetically distinct clinical Pseudomonas aeruginosa isolates from public hospitals in central Pakistan. *Journal of infection and public health*.

[25] Ahmed M. S. (2019). The investigation of molecular characterization of presumptive Listeria monocytogenes isolates from a food-processing environment. *Iranian Journal of Veterinary Research*.

[26] Bakhshi B., Afshari N., Fallah F. (2018). Enterobacterial repetitive intergenic consensus (ERIC)-PCR analysis as a reliable evidence for suspected Shigella spp. outbreaks. *Brazilian Journal of Microbiology*.

[27] Ranjbar R., Tabatabaee A., Behzadi P., Kheiri R. (2017). Enterobacterial repetitive intergenic consensus polymerase chain reaction (ERIC-PCR) genotyping of Escherichia coli strains isolated from different animal stool specimens. *Iranian journal of pathology*.

[28] Soria-Segarra C., Soria-Segarra C., Catagua-Gonzalez A., Gutierrez-Fernandez J. (2020). Carbapenemase producing Enterobacteriaceae in intensive care units in Ecuador: results from a multicenter study. *Journal of infection and public health*.

[29] Hoerr V., Duggan G. E., Zbytnuik L. (2016). Characterization and prediction of the mechanism of action of antibiotics through NMR metabolomics. *BMC Microbiology*.

[30] Chambers H. F., Kocagoz T., Sipit T., Turner J., Hopewell P. C. (1998). Activity of amoxicillin/clavulanate in patients with tuberculosis. *Clinical Infectious Diseases*.

[31] Yotsuji A., Mitsuyama J., Hori R. (1988). Mechanism of action of cephalosporins and resistance caused by decreased affinity for penicillin-binding proteins in Bacteroides fragilis. *Antimicrobial Agents and Chemotherapy*.

[32] Davis B. D. (1987). Mechanism of bactericidal action of aminoglycosides. *Microbiological Reviews*.

[33] Silver L. L. (2017). Fosfomycin: mechanism and resistance. *Cold Spring Harbor perspectives in medicine*.

[34] Miura K., Reckendorf H. K. (1967). The nitrofurans. *Progress in Medicinal Chemistry*.

[35] Then R. L. (1982). Mechanisms of resistance to trimethoprim, the sulfonamides, and trimethoprim-sulfamethoxazole. *Clinical Infectious Diseases*.

[36] Iida-Tanaka K., Tanaka T., Irino S., Nagayama A. (1986). Enhanced bactericidal action of mouse macrophages by subinhibitory concentrations of monobactams. *Journal of Antimicrobial Chemotherapy*.

[37] Zhanel G. G., Homenuik K., Nichol K. (2004). The glycylcyclines. *Drugs*.

[38] Trimble M. J., Mlynarcik P., Kolar M., Hancock R. E. (2016). Polymyxin: alternative mechanisms of action and resistance. *Cold Spring Harbor perspectives in medicine*.

[39] Spížek J., Řezanka T. (2017). Lincosamides: chemical structure, biosynthesis, mechanism of action, resistance, and applications. *Biochemical Pharmacology*.

[40] Tenson T., Lovmar M., Ehrenberg M. (2003). The mechanism of action of macrolides, lincosamides and streptogramin B reveals the nascent peptide exit path in the ribosome. *Journal of Molecular Biology*.

[41] Al-Tawfiq J. A., Rabaan A. A., Saunar J. V., Bazzi A. M. (2020). Antimicrobial resistance of gram-negative bacteria: a six-year longitudinal study in a hospital in Saudi Arabia. *Journal of infection and public health*.

[42] Meawed T. E., Ahmed S. M., Mowafy S. M., Samir G. M., Anis R. H. (2021). Bacterial and fungal ventilator associated pneumonia in critically ill COVID-19 patients during the second wave. *Journal of infection and public health*.

[43] Cockerill F. R. (2012). *Performance Standards For Antimicrobial Susceptibility Testing: Twenty-Second Informational Supplement*.

[44] Patel J. B., Cockerill F., Bradford P. A. (2015). *Performance Standards for Antimicrobial Susceptibility Testing: Twenty-Fifth Informational Supplement*.

[45] Basak S., Singh P., Rajurkar M. (2016). Multidrug resistant and extensively drug resistant bacteria: a study. *Journal of pathogens*.

[46] Vijay S., Bansal N., Rao B. K. (2021). Secondary infections in hospitalized COVID-19 patients: Indian experience. *Infection and Drug Resistance*.

[47] Wang M., Luo L., Bu H., Xia H. (2020). One case of coronavirus disease 2019 (COVID-19) in a patient co-infected by HIV with a low CD4+ T-cell count. *International Journal of Infectious Diseases*.

[48] Narazaki M., Kishimoto T. (2018). The two-faced cytokine IL-6 in host defense and diseases. *International Journal of Molecular Sciences*.

[49] Ambrosi C., Scribano D., Sarshar M., Zagaglia C., Singer B. B., Palamara A. T. (2020). Acinetobacter baumannii targets human carcinoembryonic antigen-related cell adhesion molecules (CEACAMs) for invasion of pneumocytes. *mSystems*.

[50] Chen N., Zhou M., Dong X. (2020). Epidemiological and clinical characteristics of 99 cases of 2019 novel coronavirus pneumonia in Wuhan, China: a descriptive study. *The Lancet*.

[51] Yuan W.-L., Shen Y.-J., Deng D.-Y. (2018). Sex bias of Acinetobacter baumannii nosocomial infection. *American Journal of Infection Control*.

[52] McClelland E. E., Smith J. M. (2011). Gender specific differences in the immune response to infection. *Archivum Immunologiae et Therapiae Experimentalis*.

[53] Donadu M. G., Mazzarello V., Cappuccinelli P. (2021). Relationship between the biofilm-forming capacity and antimicrobial resistance in clinical acinetobacter baumannii isolates: results from a laboratory-based in vitro study. *Microorganisms*.

[54] Shinohara D. R., dos Santos Saalfeld S. M., Martinez H. V. (2021). Outbreak of endemic carbapenem-resistant Acinetobacter baumannii in a coronavirus disease 2019 (COVID-19)–specific intensive care unit. *Infection Control & Hospital Epidemiology*.

[55] Sun W., Weingarten R. A., Xu M. (2016). Rapid antimicrobial susceptibility test for identification of new therapeutics and drug combinations against multidrug-resistant bacteria. *Emerging Microbes and Infections*.

[56] Ang H., Sun X. (2018). Risk factors for multidrug-resistant Gram‐negative bacteria infection in intensive care units: a meta‐analysis. *International Journal of Nursing Practice*.

[57] Chedid M., Waked R., Haddad E., Chetata N., Saliba G., Choucair J (2021). Antibiotics in treatment of COVID-19 complications: a review of frequency, indications, and efficacy. *Journal of Infection and Public Health*.

[58] Nikaido H. (2009). Multidrug resistance in bacteria. *Annual Review of Biochemistry*.

[59] Usai D., Donadu M., Bua A. (2019). Enhancement of antimicrobial activity of pump inhibitors associating drugs. *The Journal of Infection in Developing Countries*.

[60] Monteiro K. L. C., de Aquino T. M., Mendonça Junior F. J. B. (2020). An update on Staphylococcus aureus NorA efflux pump inhibitors. *Current Topics in Medicinal Chemistry*.

[61] Lautenbach E., Synnestvedt M., Weiner M. G. (2009). Epidemiology and impact of imipenem resistance in Acinetobacter baumannii. *Infection Control and Hospital Epidemiology*.

[62] Shin H.-S. (2020). Empirical treatment and prevention of COVID-19. *Infection and chemotherapy*.

[63] Mutlu Yilmaz E., Sunbul M., Aksoy A., Yilmaz H., Guney A. K., Guvenc T. (2012). Efficacy of tigecycline/colistin combination in a pneumonia model caused by extensively drug-resistant Acinetobacter baumannii. *International Journal of Antimicrobial Agents*.

[64] Moffatt J. H., Harper M., Harrison P. (2010). Colistin resistance in Acinetobacter baumannii is mediated by complete loss of lipopolysaccharide production. *Antimicrobial Agents and Chemotherapy*.

[65] Sun B., Liu H., Jiang Y., Shao L., Yang S., Chen D. (2020). New mutations involved in colistin resistance in Acinetobacter baumannii. *mSphere*.

[66] Goodlet K. J., Benhalima F. Z., Nailor M. D. (2019). A systematic review of single-dose aminoglycoside therapy for urinary tract infection: is it time to resurrect an old strategy?. *Antimicrobial Agents and Chemotherapy*.

[67] Ahmed S. S., Shariq A., Alsalloom A. A., Babikir I. H., Alhomoud B. N. (2019). Uropathogens and their antimicrobial resistance patterns: relationship with urinary tract infections. *International Journal of Health Sciences*.

